# Supplementary figures and images for: Evaluation and identification of stem rust resistance genes Sr2, Sr24, Sr25, Sr26, Sr31 and Sr38 in wheat lines from Gansu Province in China
Source: PeerJ. 2017 Dec 21;5:e4146. doi: 10.7717/peerj.4146 (PMC6055087; doi:10.7717/peerj.4146)

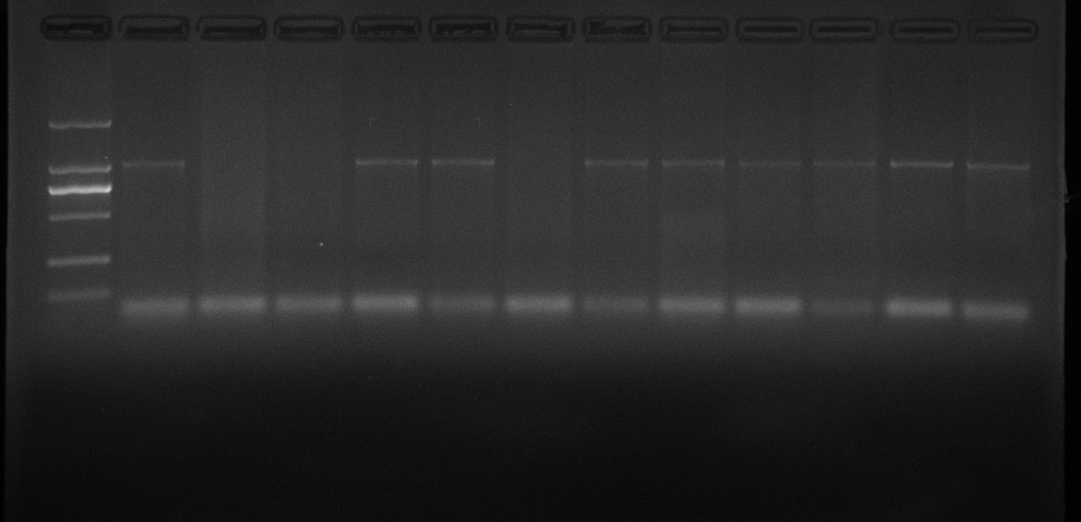

Supplement: Data S1 [file peerj-05-4146-s001.jpg]

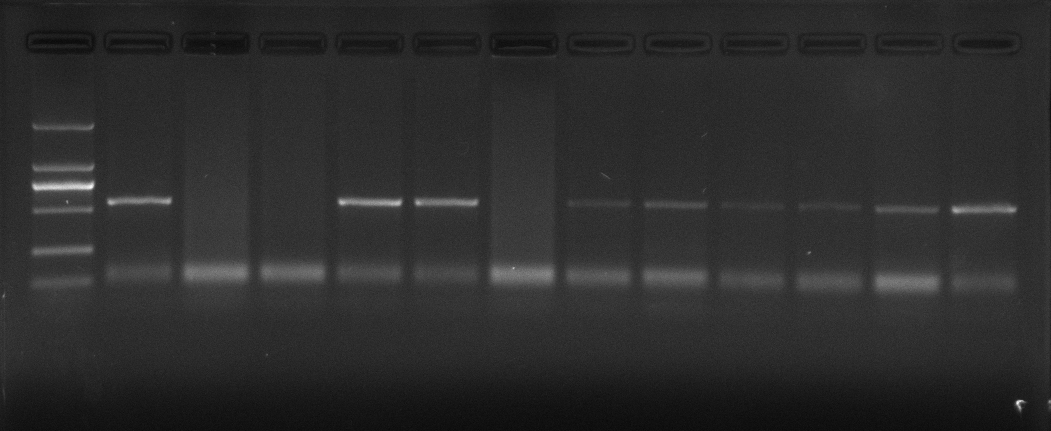

Supplement: Data S2 [file peerj-05-4146-s002.jpg]
